# Pharmacology and Potential Implications of Nicotinamide Adenine Dinucleotide Precursors

**DOI:** 10.14336/AD.2021.0523

**Published:** 2021-12-01

**Authors:** Jing She, Rui Sheng, Zheng-Hong Qin

**Affiliations:** Department of Pharmacology and Laboratory of Aging and Nervous Diseases, Jiangsu Key Laboratory of Neuropsychiatric Diseases, College of Pharmaceutical Sciences, Soochow University, Suzhou 215123, China.

**Keywords:** NAD^+^, NA, NAM, NMN, NR, aging

## Abstract

Coenzyme I (nicotinamide adenine dinucleotide, NAD^+^/NADH) and coenzyme II (nicotinamide adenine dinucleotide phosphate, NADP^+/^NADPH) are involved in various biological processes in mammalian cells. NAD^+^ is synthesised through the de novo and salvage pathways, whereas coenzyme II cannot be synthesised de novo. NAD^+^ is a precursor of coenzyme II. Although NAD^+^ is synthesised in sufficient amounts under normal conditions, shortage in its supply due to over consumption and its decreased synthesis has been observed with increasing age and under certain disease conditions. Several studies have proved that in a wide range of tissues, such as liver, skin, muscle, pancreas, and fat, the level of NAD^+^ decreases with age. However, in the brain tissue, the level of NADH gradually increases and that of NAD^+^ decreases in aged people. The ratio of NAD^+^/NADH indicates the cellular redox state. A decrease in this ratio affects the cellular anaerobic glycolysis and oxidative phosphorylation functions, which reduces the ability of cells to produce ATP. Therefore, increasing the exogenous NAD^+^ supply under certain disease conditions or in elderly people may be beneficial. Precursors of NAD^+^ have been extensively explored and have been reported to effectively increase NAD^+^ levels and possess a broad range of functions. In this review article, we discuss the pharmacokinetics and pharmacodynamics of NAD^+^ precursors.

## 1. Introduction

### 1.1 A introduction to coenzyme I and coenzyme II

Numerous research results have confirmed that nicotinamide adenine dinucleotide (NAD^+^) and nicotinamide adenine dinucleotide phosphate (NADPH) participate in mitochondrial energy and redox metabolism, reductive biosynthesis and cell signalling transduction [1-3], calcium homeostasis [4], gene expression [5], aging [6, 7], cell death [8], and other biological processes. NAD^+^ and NADPH exert preventive and protective effects in various diseases such as ischaemic stroke, cardiovascular disease, neuro-degenerative disease, and liver damage [9-13].

**Figure 1.**
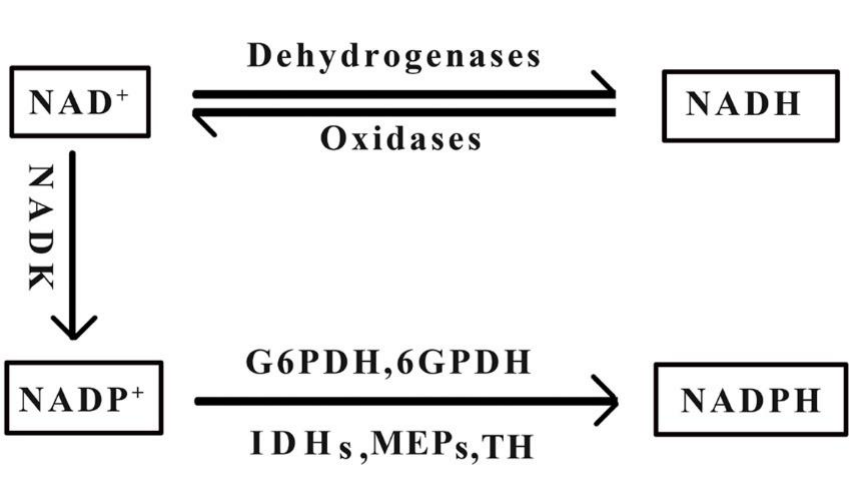
**The conversion between coenzyme I and coenzyme II.** NAD^+^: Nicotinamide adenine dinucleotide, NADH: Reduced form of nicotinamide adenine dinucleotide, NADP^+^: Nicotinamide adenine dinucleotide phosphate, NADPH: Reduced form of nicotinamide adenine dinucleotide phosphate, NADK: NAD^+^ kinase, G6PDH: Glucose-6-phosphate dehydrogenase, 6GPDH: 6 glucose phosphate dehydrogenase, IDHs: Isocitrate dehydrogenase, MEPs: Malate dehydrogenase, TH: transhydrogenase.

NAD^+^ plays a central role in the biosynthesis of NADH, NADP^+^, and NADPH, all of which require NAD^+^ as a precursor. NAD^+^ and NADH are transformed into each other under the action of NAD^+^-dependent dehydrogenase and NADH-dependent oxidase, and NAD^+^ can generate NADP^+^ under the action of NAD^+^ kinase. Under the action of glucose-6-phosphate dehydrogenase, NADPH-dependent isocitrate dehydro-genase, NADPH-dependent malate dehydrogenase, and transhydrogenase, NADP^+^ is converted to NADPH [14]. NAD^+^ is synthesised through the kynurenine pathway, Preiss-Handler pathway, and salvage pathway from tryptophan, nicotinic acid, and nicotinamide, respectively. Figure 1 depicts the conversion between coenzyme I and coenzyme II.

NAD^+^ is an essential cofactor for redox reactions and energy metabolism. NAD^+^ is also an important cofactor for NAD^+^ consuming enzymes including sirtuins, poly(ADP-ribose) polymerase (PARP) and CD38. NAD^+^ thus directly or indirectly regulates many key cellular functions, including energy metabolism, redox, DNA repair, cellular senescence and immune regulation, which are essential for maintaining metabolic homeostasis and health [15, 16]. With aging, the body's NAD^+^ content decreases [17,18]. Alteration in NAD^+^ homeostasis is found in a variety of age-related diseases, including neurodegenerative diseases, cardiovascular diseases, diabetes, and cancer [19,20]. The age-related decline in NAD^+^ is considered to be a driving force for these aging-related diseases. The level of NAD^+^ is strictly regulated by CD38 (one major NADase). However, the expression and activity of CD38 increase with aging, while inhibition or knockout of CD38 can partially prevent the decline of NAD^+^ [21,22]. During aging, senescent cells gradually accumulate in the white adipose tissue and liver. Then the inflammatory cytokines are secreted by senescent cells, the senescence-related secretory phenotype (SASP), can induce immune cells to proliferate and to express CD38, thereby consume more NAD^+^ in tissues [23,24]. These results reveal a causal relationship between cellular senescence and NAD^+^ decline during aging.

Correspondingly, to increase intracellular NAD^+^ can prevent age-related metabolic decline [25], improve the function of mitochondria and stem cells [26], maintain skeletal muscle function and exercise capacity [27]. Therefore, elevation of NAD^+^ may slow down or even reverse the progression of many aging-related diseases such as neurodegenerative diseases [28], metabolic dysfunction [29-31], immune disorders [32], mitochondrial dysfunction [33], and vascular aging [34], and extend the life span of animals [26,35-37].

However, several studies have found that the oral administration of NAD^+^ cannot effectively increase the level of NAD^+^ in plasma or in tissues. On one hand, the intestinal effect of NAD^+^ lowers its bioavailability; on the other hand, the excessively large polarity of NAD^+^ inhibits its passive transport through the plasma membrane. Therefore, the direct absorption of NAD^+^ by cells is believed to be unfeasible; however, this viewpoint may be challenged because a NAD^+^ transporter has been recently identified [38, 39]. Furthermore, the direct administration of high doses of NAD^+^ can cause insomnia, fatigue, anxiety, and other adverse reactions [40]. NAD^+^ levels in plasma or tissues do not increase significantly after oral administration of NADH mainly because orally administered NADH cannot be oxidised to NAD^+^, which inhibits its effective absorption in the intestine, although a NADH transporter has also been identified [41]; another possible reason is that NADH, before being absorbed by the gastrointestinal system in the human body, is transformed into a product that cannot produce NAM [42, 43]. Currently, intravenous infusion of NAD^+^ is the only clinically recognised method to increase the level of NAD^+^ in humans [44].

**Figure 2.**
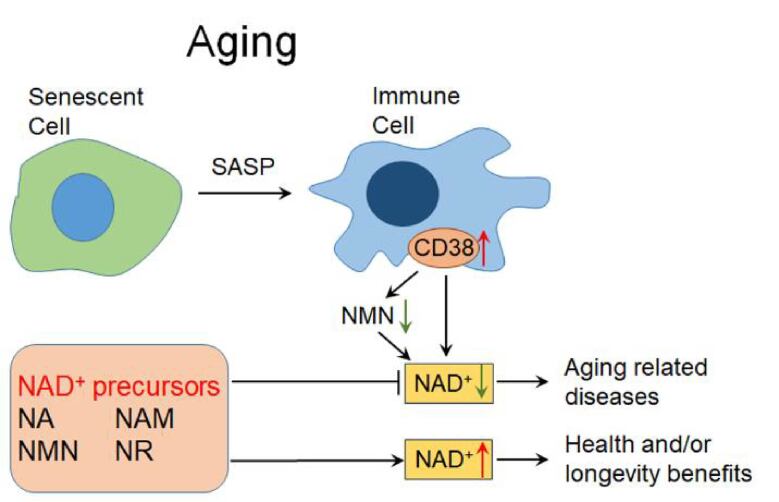
**NAD^+^ and NAD^+^ precursors in aging.** During aging, the inflammatory cytokines secreted by senescent cells, the senescence-related secretory phenotype (SASP), can induce immune cells to proliferate and to express CD38, thereby decreasing NMN and NAD^+^ in tissues [23, 24]. The age-related decline in NAD^+^ may be a driving force for aging-related diseases. The NAD^+^ precursors may have potential health and longevity benefits by increasing NAD^+^, and may be a promising strategy for alleviating aging-related diseases. NAD^+^: Nicotinamide adenine dinucleotide, NA: Nicotinic acid, NAM: Nicotinamide, NMN: Nicotinamide mononucleotide, NR: Nicotinamide riboside.

In recent years, more and more researchers have turned their attention to NAD^+^ precursors, namely nicotinic acid (NA), nicotinamide (NAM), nicotinamide mononucleotide (NMN), and nicotinamide ribose (NR). These precursors may have potential health and/or longevity benefits by increasing the level of NAD^+^ in the body and may be a promising strategy for alleviating aging-related diseases (Fig. 2).

### 1.2 NAD^+^ and NAD^+^ precursors

Tryptophan (Trp) in the de novo pathway and NA and NAM in the salvage pathway were identified after the outbreak of a deadly disease named pellagra. In the last century, this disease was common in underdeveloped countries, such as South Africa, and some rural areas in the southern United States, where the regular diet lacks NAD^+^ precursors in terms of both quantity and quality [45, 46]. The number of cases currently is relatively small and those rare cases are often detected in chronic alcoholics [47]. The study on pellagra in South Africa was conducted for the first time by Cluver EH et al. [45] and later by several other researchers. They found that pellagra is a syndrome caused by a lack of dietary tryptophan and NA in acid and amide forms [48]. The clinical signs of this disease include characteristic dermatitis, abnormal changes in the gastrointestinal tract and nervous system [49], obvious dark-pigmented rash, dermatitis, diarrhoea, and dementia [50]. The disease is pathogenic that is caused by a long-term lack of Trp in diet and can progress rapidly within 60 days. In 1937, a Professor of biochemistry first discovered the anti-pellagragenic effect of NA and NAM [51], and subsequent biochemical studies have found that after chronic immune activation, the lack of Trp in diet and inhibition of quinolinic acid phosphoryl transfer (QRPT) decrease the availability of NAD^+^, which is related to the development of pellagra [52, 53]. For the treatment of this disease, a diet rich in NA and NAM and comprising corn, eggs, cured meat, and milk was suggested, which was found to prevent the occurrence of pellagra to some extent [54]. NMN and NR have been recently identified as the precursors of NAD^+^ and have received considerable attention because of their potential therapeutic effects and fewer side effects compared with those of NA and NAM. The discovery of these two precursors also prompted researchers to further investigate the function of NAD^+^.

**Figure 3.**
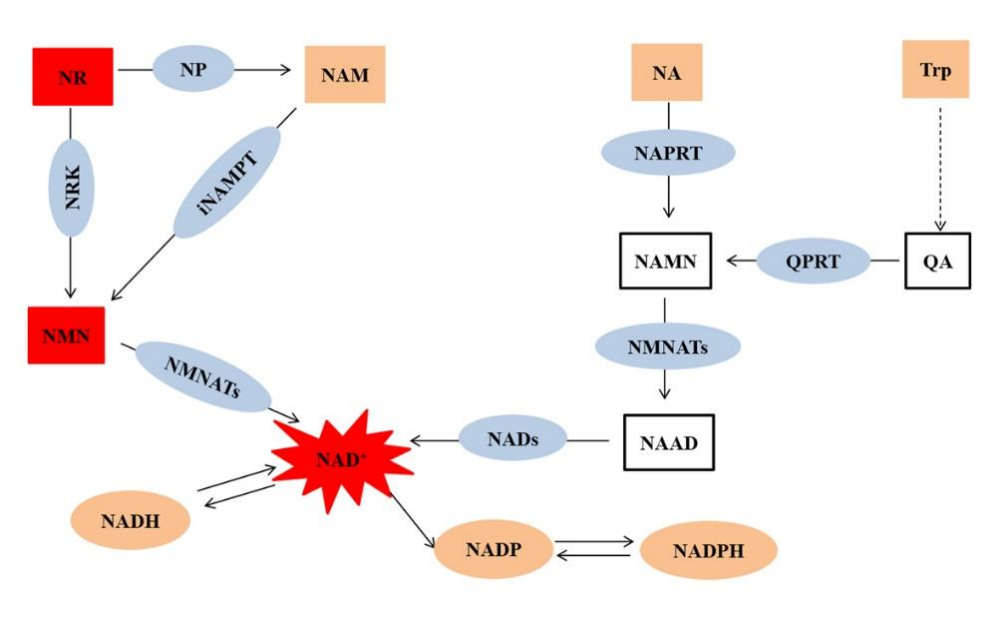
**NAD^+^ can be synthesised through three pathways: de novo, Preiss-Handler, and salvage pathways.** Trp: Tryptophan, QA: Quinolinic acid, QRPT: Quinolinic acid phosphoribosyl transferase, NAMN: Nicotinic acid mononucleotide, NAAD: Nicotinic acid adenine dinucleotide, NADs: NAD^+^ synthetase, NAD^+^: Nicotinamide adenine dinucleotide, NR: Nicotinamide riboside, NP: Nucleoside phosphorylase, NA: Nicotinic acid, NARPT: Nicotinic acid phosphoribosyl transferase, NAM: Nicotinamide, iNAMPT: Intracellular Nicotinamide phosphoribosyl transferase, NMN: Nicotinamide mononucleotide, NMNATs: Nicotinamide mononucleotide adenylyl-transferases, NRK: Nicotinamide ribose kinases, NADH: Reduced form of nicotinamide adenine dinucleotide, NADP^+^: Nicotinamide adenine dinucleotide phosphate, NADPH: Reduced form of nicotinamide adenine dinucleotide phosphate.

The three biosynthetic pathways of NAD^+^, namely the de novo pathway (Trp), Preiss-Handler pathway (NA) and salvage pathway (NA, NNM, NR), are illustrated in Figure 3. In the de novo pathway, Trp undergoes a series of reactions in eight steps to generate NAD^+^. Trp, as a precursor, first produces quinolinic acid (QA) through a five-step reaction, and one of these five-step reaction steps requires NADPH-dependent enzyme 3-Hydro-xykynurenine (3-HK); the activity of 3-HK was reported to reduce under hyperthyroidism conditions [55]. This enzyme can pass through the blood-brain barrier, which leads to the production of free radicals and vasodilation [56]. Subsequently, the generated QA produces nicotinic acid mononucleotide (NAMN) under the action of quinolinic acid phosphoribosyl transferase (QPRT). QPRT is the most critical rate-limiting enzyme in the de novo pathway. This reaction step is catalysed by the enzyme in an ATP-dependent manner and requires the participation of Mg^2+^ and 5-phosphoribosyl-1-pyro-phosphate (PRPP). Finally, the generated NAMN is transformed to NAD^+^ under the action of nicotinamide mononucleotide adenylyl-transferases (NMNATs) and NAD^+^ synthetase (NADs) in a two-step reaction, and these two steps require 2 ATP molecules.

In the Preiss-Handler pathway, NA as a precursor undergoes a total of three reactions to generate NAD^+^. First, NAMN is generated under the action of nicotinic acid phosphoribosyl transferase (NAPRT). The reaction catalysed by NAPRT is ATP-dependent and requires the participation of PRPP. Subsequently, the generated NAMN is converted to NAD^+^ under the action of NMNATs and NAD^+^ synthetase in a two-step reaction, and this reaction also consumes 2 ATP molecules.

In the salvage pathway, NAM serves as the precursor and undergoes a two-step reaction to generate NAD^+^. First, it is converted to NMN under the action of nicotinamide phosphoribosyl transferase (NAMRT). The reaction catalysed by NAMPT is also ATP-dependent and requires PRPP. The generated NMN is converted to NAD^+^ under the action of NMNATs, and the reaction requires 1 ATP molecule. NR, as the precursor of the additional NAD^+^ salvage pathway, can generate NAD^+^ through two pathways: first, under the action of nucleoside phosphorylase (NP), NR produces NAM and after 3 steps generates NAD^+^; second, NR generates NAD^+^ through a 2-step reaction under the action of nicotinamide ribose kinases (NRK).

These precursors require different amounts of ATP to convert into NAD^+^; Trp, NA, NAM, NR, and NMN require 4 ATP, 3 ATP, 2 ATP, 2 ATP, and 1 ATP molecule, respectively. The synthesis of NAD^+^ through the de novo pathway is a long pathway, which consumes more energy than the salvage pathway. Trp is far less effective in increasing the concentration of NAD^+^ compared with other precursors; daily administration of 15 mg/kg of NA or NAM can be used to prevent and treat pellagra; however, the administration of 60 times or higher amount of Trp can produce similar effects as those of NA and NAM [57].

## 2. Pharmacokinetics of NAD^+^ precursors

### 2.1 Cell entry modes of NAD^+^ and its precursors

Current knowledge suggests that all precursors and NAD^+^ must enter cells to produce biochemical and physiological actions. Increasing evidence indicates that NAD^+^ cannot enter cells directly through the plasma membrane and that it must be converted into smaller, less charged molecules to enter cells [58]. NAD^+^ can be degraded into NAM by membrane-bound CD38 and CD157 outside the cells, and the produced NAM can further produce NMN under the action of extracellular nicotinamide phosphoribosyl transferase (ENAMPT); NAD^+^ can also generate NMN directly under the action of membrane-bound CD73 outside the cells [59-61]. Some studies have identified a special NAD^+^ transporter, the connexin 43 (Cx43) channel, through which NAD^+^ can enter cells. Cx43 is highly expressed in cardiomyocytes [62, 63]. NADH has been reported to enter cells through the P2X7 receptor [41]; however, the same has not been confirmed by other researchers. Further research is required to determine the cell-specific efficiency of NAD^+^/NADH transporters and other NAD^+^/NADH transporters, if any, in addition to the Cx43 and P2X7 channels.

In the de novo pathway, Trp enters cells through carrier proteins (SLC7A5 and SLC36A4), which transport large, neutral amino acids [64]. NA and NAM, two forms of vitamin B3, can directly pass through the plasma membrane. Of these forms, the entry of NA into cells is mediated by a membrane carrier system, which includes a pH-dependent anion antiporter and a proton co-transporter (SLC5A8 or SLC22A13) [65, 66]. NAM can enter cells through two pathways; it may either be directly transported into the cell in its intact form or be converted into a metabolite of the salvage pathway and taken up by cells. The presence of the enzyme NAMPT, which converts NAM to NMN both inside and outside the cells, indicates that both pathways are feasible [67, 68]. Relevant studies on rodents have reported that NAM can be directly absorbed by the intestine [69]. The third form of vitamin B3, NR, does not require conversion to enter cells, which accounts for the high bioavailability of NR. NR enters cells through equilibrative nucleoside transporters (ENTs) and is phosphorylated into NMN by nicotinamide ribose kinase (NRK1/2) in cells [70, 71].

**Figure 4.**
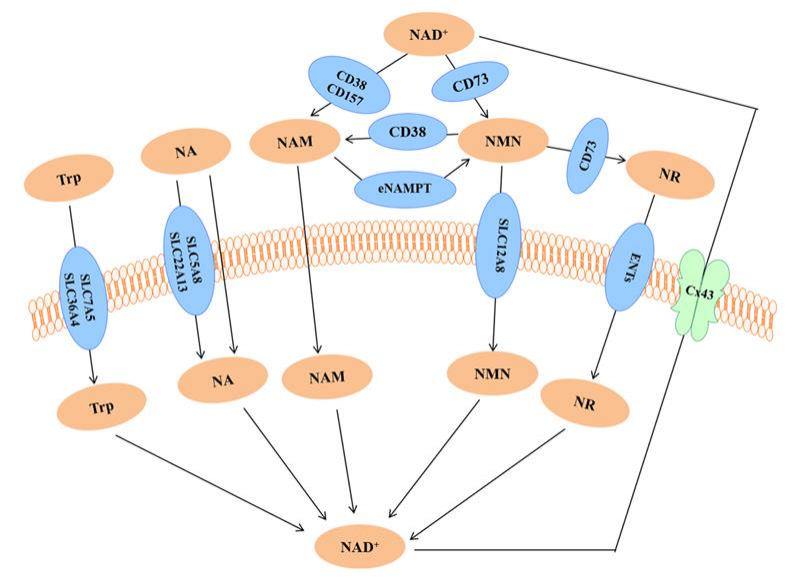
The routes of NAD^+^ and its precursors to enter cells.

The routes through which NMN enters cells are described in literature as being highly complicated. First, NMN is transformed into NAM under the action of membrane-bound CD38, and then, it directly passes through the plasma membrane [21]; second, under the action of membrane-bound CD73, NMN is transformed into NR, which enters cells through ENTs [72]; third, NMN can directly enter cells through an NMN-specific transporter, a recently discovered transporter, which is highly expressed in the small intestine and encoded by the Slc12a8 gene [73]. Therefore, the uptake of NMN may be cell- or tissue-specific. However, a recent study in yeast showed that dephosphorylation of NMN into NR is necessary for the production of NAD^+^, whereas another study reported that the conversion of NMN to NAD^+^ can be inhibited by silencing the gene CD73, indicating that NMN must be converted into NR [4, 69, 70]. Therefore, investigating the uptake mode and kinetics of cell- or tissue-specific NAD^+^ precursors is essential. Figure 4 illustrates the routes through which NAD^+^ and its precursors enter cells.

### 2.2. Pharmacokinetics of NAD^+^ precursors

To maintain the level of NAD^+^ in vivo, most of NAD^+^ is synthesised through the salvage synthesis pathway rather than the de novo pathway. Trp can produce kynurenic acid and serotonin in addition to producing QA for further synthesis of NAD^+^ [74]. Trp is considered to be the main precursor of NAD^+^ production in the liver [75]. NA and NAM are the only precursors that are increased in the liver 15 min after oral administration of NA or NAM, suggesting that the liver can use both de novo and salvage pathways to synthesis NAD^+^ [76].

We hereafter discuss the pharmacokinetics of NA and NAM. At high doses, the half-life of NA is 1 h, whereas that of NAM is 4 h. Studies have shown that the administration of high doses of NA will increase the level of NAM, but whether the administration of high doses of NAM influences the content of NA is still unclear [77]. However, the administration of NAM has been reported to cause skin flushing, which is a common adverse reaction that occurs after the administration of NA, indicating that the administration of high doses of NAM may also increase the NA content [78].

In a study, the ability of NA and NAM to increase NAD^+^ was compared by orally administering NAD^+^ precursors to mice and NA was reported to produce the lowest level of NAD^+^ [76]. Oral administration of NA has been shown to result in a two-fold increase in NAD^+^ levels in the liver along with an increase in the NAAD level [76, 79]. In another study, 30 mg/kg NA and 4000 mg/kg NA were administered to rats, and 4000 mg/kg NA was found to increase the level of NAD^+^ in the bone marrow of rats [79]. In a recent clinical study, after 10 or 4 months of administration of NA (750-1,000 mg/day), the blood and muscle NAD^+^ levels of human subjects were significantly increased. NA can also alleviate systemic NAD^+^ deficiency and improve muscle performance in adult-onset mitochondrial myopathy [80]. In another study, six healthy male subjects who took the upper level of NAM that can be tolerated per day (200 mg) in a single oral administration caused the maximum NAM blood concentration to increase by 30 times at 0.5 h, and then continued to decrease until 6 h, and the NAD^+^ blood concentration also increased significantly with the maximum concentration at 12 h [81]. The pharmaco-kinetics of oral administration of 3-6 g NAM in humans has been studied, and high doses have been shown to produce adverse reactions such as nausea and vomiting [82]. Although the capacity of NAM to increase NAD^+^ levels offer an advantage over NA, the less accumulation of ADP-ribose (ADPR) mediated by NAM also indicates a disadvantage. ADPR is a marker for NAD^+^-consuming enzymes activities [76], and NAM inhibits the activities of NAD^+^-consuming enzymes, such as PARP and sirtuin.

Although limited pharmacokinetic data are available on NMN and NR compared with those on NA and NAM, a few studies have demonstrated that NMN and NR can effectively increase the NAD^+^ content in various tissues. Limited evidence shows that the administration of NMN can enhance NAD^+^ levels in various peripheral tissues such as pancreas [29], liver [83], adipose tissue [84], heart [85], skeletal muscle [33], and kidney [86]. Reports also indicate that after the administration of NMN, NAD^+^ levels in the testes [87] and eyes [25] are significantly increased. Furthermore, NMN has been reported to rapidly increase the level of NAD^+^ in the hippocampus, hypothalamus, and other brain regions within 15 min of intraperitoneal administration [88, 89], which further suggests that NMN can pass through the blood-brain barrier, thereby contributing to the biosynthesis of NAD^+^ in the brain. Studies have reported that NMN can be detected in the mouse plasma, liver, adipose tissue, and pancreas within 15 min of the administration of 500 mg/kg NMN to wild-type mice through intraperitoneal injection; NMN is then used for NAD^+^ biosynthesis, which increases the level of NAD^+^ in the liver by 2-3 times [29]. A study also reported that the administration of 300 mg/kg NMN to mice through gavage increases the plasma NMN level significantly within 2.5 min and further increases the level after 10 min; however, the plasma NMN level returned to the original level within 15 min. Simultaneously, an increase in NAD^+^ levels in the liver, skeletal muscle, and cerebral cortex was observed. Results of the study indicated that NMN reaches the blood circulation from the intestine within 2-3 min and reaches the tissue from the blood circulation within 15 min [25]. Another study reported that the retention time of NMN in the body after intraperitoneal injection may be longer than that of NAM [90]. Some studies have shown that the plasma NAM content and hippocampal NR level are significantly increased after NMN injection, suggesting that at least a part of NMN is transformed into NAM and NR [40, 91]. In a recent clinical study, after a single oral administration of 100-500 mg of NMN in 10 healthy men, the plasma concentrations of NMN and NAD^+^ metabolites (N-methyl-2-pyridone-5-carboxamide and N-methyl-4-pyridone-5-carboxamide) increased significantly [92]. NMN is generally believed to show good chemical stability. More than 90% of NMN can remain stable for 7-10 days in drinking water at room temperature [25]. NMN is also relatively stable in human HEK293 cell culture in FBS-free medium, and only 5% of NMN is dephosphorylated to NR by cells in 24 h [93]. However, during aging, senescence-induced inflammation promotes the accumulation of CD38 in immune cells. Then CD38 degrades the extracellular NMN through its ecto-enzyme activity, resulting in the decrease of intracellular NAD^+^ [24]. Trammell et al. administered NR orally to study its effect on human peripheral blood mononuclear cells (PBMCs) and mouse liver NAD^+^ metabolism [76]; results of the study showed that the concentrations of all NAD^+^ metabolites, except NAM, were elevated in PBMCs. Moreover, the concentration of NAAD, a metabolite that is supposed to increase after the administration of NA instead of NR [94], was also assessed, which was found to be significantly increased, although a slight delay in the increase in the concentration of NAAD was observed relative to other metabolites, NAAD may be a biomarker of NAD^+^ biosynthesis and indicate the conversion of NR to NAD+ over time. In the liver of mice, the oral administration of 185 mg/kg NR increased the levels of NAM and NAD+ by approximately four times. Similarly, the level of NAD+ and NAAD in blood cells of a healthy 52-year-old man who took NR (1000 mg/kg) for 7 days was found to increase by 2.7 times. The study also found that the ability of NR to increase ADPR is 2-3 times that of NAM and that ADPR is a marker of the activity of NAD+-depletion enzymes such as sirtuin [76].

The pharmacokinetics of NMN and NR have not been fully studied. NMN and NR exhibit better pharmacological properties compared with NA and NAM; however, a deeper dosage and mechanistic research are required to compare pharmacokinetics between NMN and NR. Both NMN and NR undergo primary metabolism before being absorbed in the body and are rapidly converted into intermediates [27]. Exploration of the pharmacokinetics of NMN and NR in the body can help determine their optimal concentration for different applications and provide insights into their pharmacological mechanisms of action.

## 3. Pharmacological actions of NAD^+^ precursors

NAD^+^ precursors are widely present in natural foods such as meat, eggs, dairy products, and whole wheat [25, 95]. NA is produced in plants and algae; NAM is the main form of vitamin B3 that can be absorbed from foods, and it is also a byproduct of deacetylation and ADP-ribosylation mediated by NAD^+^-metabolising enzymes such as SIRT, PARP, and CD38. NMN and NR are found in vegetables (such as broccoli and cucumber), fruits (such as avocados), and meats (such as beef) [96]. NR, the third discovered NAD^+^ precursor, is naturally present in milk and is considered a nutritious food source [97]. Several studies have shown that relying more on nutritious plant foods rather than meat may be the most effective strategy for obtaining health benefits and extending the lifespan [98].

Both NA and NAM are the forms of vitamin B3 that were introduced more than 50 years ago for the prevention and treatment of pellagra. Usually, 15 mg/day of NA, the acidic form of niacin, is commonly used in clinics to treat hyperlipidemia [44]. Intake of 1-3 g of NA per day has been reported to effectively regulate the ratio of low-density lipoprotein to high-density lipoprotein (LDL: HDL) [99, 100]. Similarly, increased NA levels have been shown to improve the genome integrity, and NA deficiency has been shown to cause chromosomal instability [101, 102]. High-dose NAM, the amide form of niacin, is used in radiotherapy and chemotherapy to promote microvascular blood flow in the brain [103, 104]. Studies have also reported that in several types of animal models of diabetes, NAM can prevent and reduce the progression of diabetes [105, 106]. NAM has been shown to inhibit cell apoptosis caused by glutamate-induced excitotoxicity [107, 108]. Moreover, it has been reported to maintain the genomic stability and reduce the incidence of skin cancer [109]. NAM can also improve remyelination after stroke [110]. In addition, NAM is widely used to treat skin diseases including autoimmune vesicular diseases.

Researchers have paid considerable attention to the precursors NMN and NR in recent years because these are highly efficient in increasing NAD^+^ levels. These two precursors are involved in the DNA repair and ATP production and also play roles in cell signal transmission [98]. NMN was reported to improve insulin sensitivity and exert a positive effect on insulin levels [111]. Some studies have reported that NMN may be an effective intervention for patients with hypoglycaemia [112]. NMN participates in mitochondrial energy metabolism by improving mitochondrial respiration. NMN also has a hepatoprotective effect. NMN supplementation can prevent liver fibrosis by promoting the degradation of prostaglandin E2 and inhibiting the activation of hepatic stellate cells [113]. The application of NMN alone can restore the cardiac systolic function of elderly mice, while the combined application of NMN and SS-31 (a drug that targets mitochondria) in mice improved both systolic and diastolic function, and reduced myocardial hypertrophy [114]. Studies have shown that NMN can improve cognitive impairment in the Alzheimer disease (AD) mice model [115]. NMN can also improve the depressive behavior in animal models [116] and improve the survival rate in the Parkinson disease (PD) model in vitro [117]. NMN has also been reported to exert a protective effect on secondary brain damage caused by cerebral haemorrhage [118], haemorrhagic transformation in the MCAO (middle cerebral artery occlusion) model [119] and haemorrhagic transformation caused by ischaemic stroke with tPA [120]. Additionally, NMN protects against cardiac ischaemia and ischaemic stroke [121, 122], Therefore, NMN may be a potential drug for the treatment of age-related neurodegenerative diseases. Low doses of NMN were shown to improve the quality of female oocytes [123], thereby improving female fertility. Because NMN can effectively improve the quality of aging oocytes, NMN may be a potential drug for the treatment of fertility problems in older women [123-126]. Studies have shown that NMN can reverse age-related weight gain and cognitive impairment [25, 116]. Moreover, administration of NMN in aging mice has been shown to improve vascular oxidative stress and energy metabolism, restore the activity of sirtuin, and reverse age-related arterial dysfunction [29, 127]. NMN also improved the impaired neurovascular coupling response in the aged cortex and the resulting vascular cognitive impairment by the induction of genes involved in mitochondrial regeneration, anti-inflammation and anti-apoptosis [128]. All these initial studies suggest that NMN exhibits certain therapeutic prospects in aging-related diseases.

NR is the main precursor of NAD^+^ in the central nervous system and the preferred precursor in mitochondria. It maintains the function of mitochondria by regulating the activity of sirtuin [44]. NR is also the preferred precursor for supplementing NAD^+^ levels in animal models of heart failure [129] and was shown to reduce cholesterol in obese mice [130]. It has also been shown to exert a certain ameliorating effect on alcohol-induced liver disease and depressive behaviour [28, 131, 132] and improve diabetic lesions and hepatic steatosis in mice with high-fat diet-induced obesity [133, 134]. NR can also ameliorate angiotensin Ⅱ-induced cerebral small vessel disease in mice [135] and prevent noise-induced hearing loss [132, 136]. Similar to NMN, NR can also improve female fertility [137, 138]. NR is the only precursor that can prevent axon degeneration [139] as well as the oxidative stress and organ damage caused by sepsis [140]. Moreover, NR has been shown to exert a certain degree of therapeutic effect in the pathological progress of neurodegenerative diseases such as AD [28, 141], PD [142], aging [138, 143], cerebral apoplexy [129], and hypertension and cardiovascular diseases [76, 129, 144]. Numerous studies have shown that NR can increase the lifespan of all species tested so far, including mice [35, 37, 145].

**Figure 5.**
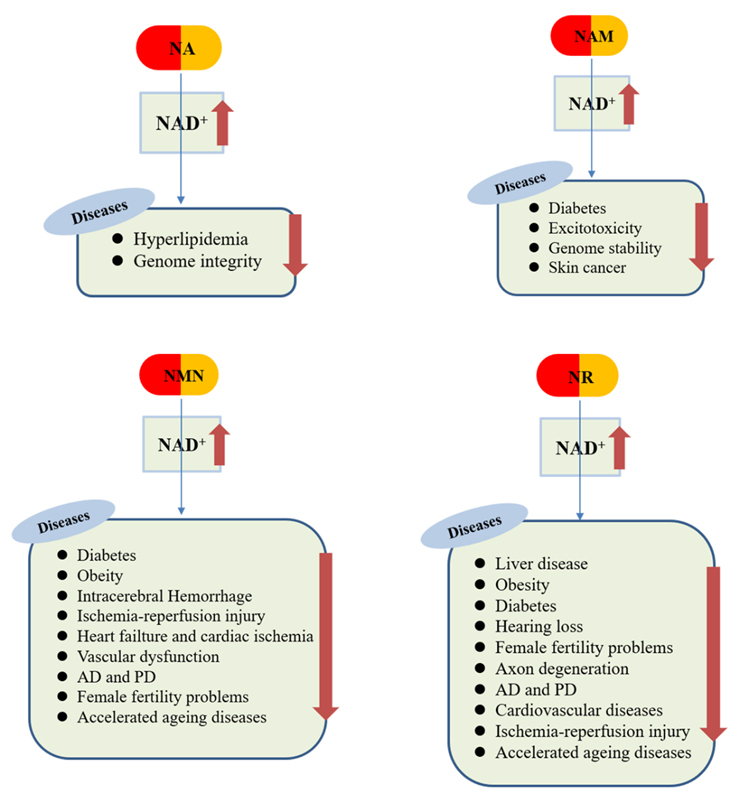
The pharmacological actions of NAD^+^ precursors.

Although NR, NMN, NA, and NAM can effectively improve NAD^+^ levels, many issues still remain to be explored. As an NAD^+^ precursor enters the body, it is converted into NAD^+^, which is further converted into NADH, NADP^+^, and NADPH. Therefore, whether the beneficial effects are produced by the precursor itself or the transformed NAD^+^ or other coenzymes is unclear. Canto et al. reported that the ability of NA to lower cholesterol levels can be attributed to its ability to increase NAD^+^ levels [130]. NAD^+^ depletion increases the skin’s sensitivity to ultraviolet light, increases the DNA damage response, and eventually increases the instability of the genome and incidence of skin cancer. Conversely, NAM increases the genomic stability and decreases skin cancer incidence, which can also be attributed to its ability to increase NAD^+^ levels. Moreover, the administration of NA and NAM did not show the same physiological results compared with those of NMN and NR [76, 95, 146]. Liver damage caused by diet can be reversed by endogenous metabolism of NR rather than NAM [147]. Oral administration of NR can significantly improve the survival rate of immune-deficient mice and regeneration of haematopoietic stem cells, which cannot be achieved by administrating NA or NAM [148]. The effects that are observed after the administration of precursors may not necessarily be produced by NAD^+^, and different functions of the precursor itself or its transformation into other coenzymes may also play a role (Fig. 5).

## 4. Safety and side effects of NAD^+^ precursors

Studies have found that with aging, the body's intake of L-Trp [149, 150] and the de novo synthesis of NAD^+^ decrease. Among all organs of the body, only the liver contains all the synthetases involved in the de novo pathway, including the rate-limiting enzyme QPRT. Studies have shown that most Trp is consumed in the liver when the body is not aging [151]. However, the absence of QPRT has no significant effect on NAD^+^ levels in tissues, including the liver [152]. These results indicate that mammals may synthesise NAD^+^ mainly through the salvage synthesis pathway. The de novo pathway synthesises two basic neurotransmitters, namely glutamate and acetylcholine, in addition to generating NAD^+^. Some intermediates are also produced that regulate the activity of N-methyl-D-aspartic acid (NMDA) [153], for example, the antagonist of NMDA receptor kynurenic acid exerts a protective effect, and the agonist of NMDA receptor QA induces excitatory toxicity through glutamate receptors; hence, the de novo pathway is a double-edged sword that regulates the neuronal function [12].

NA has been clinically used to treat dyslipidemia because it can lower blood lipid levels. However, the dosage of NA should be carefully used. If the daily dosage exceeds 50 mg, it will not only cause headaches and dizziness but also induce the production of prostaglandins, cause irritation to skin immune cells, and dilate skin capillaries, leading to skin flushing and itching [154]. Studies have found that this side effect is due to the activation of the G protein-coupled receptor, GPR109A (HM74A). NA can also cause spontaneous skin flushing reactions even at therapeutic doses because it acts as an agonist of this G protein-coupled receptor [155, 156], and this side effect greatly limits its clinical application [157, 158]. In addition, some of the animal studies have used a much higher NA dosage than those used in clinical patients to assess the effects of NA. For example, NA improves the neuronal function after hypoxic injury at a concentration of 250 μM-1000 μM in the culture medium, which exceeds the usual therapeutic concentration achievable in humans [159, 160]. Therefore, an optimal dosage of NA required for the elevation of NAD^+^ in the human body should be investigated. In recent years, the results of various clinical studies on NA and NAM have shown that NAM is safer and more easily absorbed by the gastrointestinal tract than NA; although NAM reaches a serum peak 1 h after its oral administration, high doses of NAM can cause adverse reactions such as nausea and vomiting [161]. As mentioned earlier, NAM is a byproduct of NAD^+^ catabolism and a natural feedback inhibitor of NAD^+^-dependent enzymes such as sirtuin. Several studies have shown that the activities of PARP, sirtuin, and CD38 are inhibited at high doses of NAM [162]. The inhibition of NAD^+^-dependent enzymes produces side effects in the body. For example, a study demonstrated that the administration of NAM increases the accumulation of liver fat in a rat model of choline deficiency [44]. In addition, NAM consumes methyl groups and leads to a decrease in epigenetic methylation [163, 164]. Therefore, NAM is not considered to be an ideal precursor for supplementing NAD^+^ due to its feedback inhibition of NAD^+^-dependent enzymes and side effects of methyl depletion [165]. Considering the side effects of NA and NAM, neither NA nor NAM are the ideal precursors for increasing NAD^+^ levels.

According to reports, low-dose NMN may be effective and safe. Single oral administration of 500 mg of NMN in healthy individuals is also safe and does not cause adverse reactions [92]. Following the oral administration of 300 mg/kg of NMN to normal wild-type mice (C57BL/6) for up to one year, the mice did not display any harmful or toxic effects, suggesting the superior safety and tolerability of NMN [25]. However, high-dose NMN may have adverse effects. Although low-dose NMN can improve the quality of female oocytes, high-dose NMN can reduce sperm quality [166]. In particular, the brain is highly sensitive to NMN, and high doses of NMN may exert adverse effects on neurons after ischaemia [121]. Studies have shown that high doses of NMN promote axonal degeneration in case of nerve damage [167-170]. NMN may also exacerbate in vitro axonal degeneration caused by a chemotherapy drug, vincristine [171]. Although numerous studies have proved the potential of NMN in the treatment of metabolic and aging-related diseases, its toxicological and clinical effects have not been sufficiently studied, and further studies are required to investigate the optimum dose range of NMN and long-term safety to humans.

Clinical studies on the short-term and long-term administration of NR have demonstrated the superior bioavailability and safety of NR. It is considered safe even when administered at a dose of 2000 mg a day for 12 weeks, and no adverse symptoms, such as nausea and vomiting, or undesirable skin flushing have been reported [143, 172]. Supplementation of NR neither inhibits NAD^+^-dependent enzymes nor causes side effects such as liver damage [76]. A study indicated that with an increase in the NR level in tissues following NR administration, the activity of the enzyme sirtuin is significantly increased compared with NAM administration [76]. Compared with other precursors, NR is gradually becoming a preferred candidate precursor because of its high bioavailability, safety, and ability to increase NAD^+^ levels. It offers many potential health benefits in diseases such as cardiovascular diseases [173, 174], neurodegenerative diseases [130, 175, 176], and metabolic diseases [177]. In summary, NR is a more effective precursor for synthesising NAD^+^ and increasing the activity of NAD^+^-dependent enzymes than NA and NAM. However, further research is required to explore whether NR can cause skin flushing or other adverse symptoms. Although NR produces no serious adverse reactions, it has not been shown to improve insulin sensitivity, endogenous glucose production, glucose disposal, and oxidation [172, 178]. Therefore, further studies are required to determine the benefits of NR.

NAD^+^ participates in thousands of biochemical reactions in the body and thus maintains and regulates various physiological processes such as DNA repair, calcium homeostasis, and energy metabolism. Whether the supplementation of NAD^+^ precursors to increase the NAD^+^ content produces, in addition to the aforementioned effects, other side effects, especially diseases involving cell proliferation, such as tumours and atherosclerotic plaques, remains unclear. Energy metabolism not only plays an important role in the growth of normal cells but also promotes the growth of tumour cells. Moreover, aerobic glycolysis and other energy metabolism pathways in tumour cells are abnormally upregulated, thus generating a large amount of energy and metabolic intermediates to satisfy the rapid proliferation of tumour cells [179]. In addition, NAD^+^ not only acts a key coenzyme in aerobic glycolysis but also plays a central role in other energy metabolism pathways, including the TCA cycle [16]. Tumour cells have higher NAD^+^ levels than normal cells; therefore, NAD^+^ poses a risk of driving the growth of tumours. For chemotherapy drugs under development, some researchers have turned their attention to drugs that can consume NAD^+^ [180]. The consumption of NAD^+^ in tumour cells inhibits the ability of NAD^+^ to repair DNA and participate in energy metabolism, thereby inhibiting the rapid proliferation of tumour cells. The consumption of NAD^+^ also promotes the production of reactive oxygen species, which in turn causes the disruption of tumour cells due to autophagy and apoptosis [181]. Inhibition of the rate-limiting enzyme NAMPT in the salvage synthesis pathway in tumour cells and animal tumour models has been shown to reduce the growth of tumour cells and enhance survival of animals [182-184]. However, some researchers believe that the decline in NAD^+^ levels may be related to aging-related diseases including tumours. Lack of NA in rats along with carcinogen exposure has been shown to increase the incidence of tumours [185, 186]. Moreover, the incidence of skin tumours in mice was shown to reduce with the topical application of NAM or supplementation of NA in the diet [187]. Recently, NR was reported to reduce the proliferation and activation of liver progenitor cells involved in liver tumour heterogeneity [188]. NR treatment can also reduce the size of the established liver tumour [189]. Increasing NAD^+^ levels have been shown to play an important role in the prevention of liver cancer and pancreatic cancer in mice [188, 190, 191]. The expression of CD38 increases with cell aging, thereby degrading NMN, which is one of the main reasons for the decline in NAD^+^ levels in senescent cells. According to a study, the proliferation of gliomas can be inhibited by inhibiting CD38, thereby prolonging the survival time of glioma mice [192]. Daratumumab, a CD38 monoclonal antibody, is a drug used for the treatment of multiple myeloma [193].

## 5. Potential clinical applications and future study

The first discovered NAD^+^ precursors, NA and NAM, are used as both food supplements and drugs. Although they are not as effective as NR and MNM in increasing NAD^+^ levels, they are relatively cheaper. However, the reason for these precursors being not much sought-after supplements for NAD^+^ remain to be investigated. NMN and NR may be favourable precursors for increasing the level of NAD^+^ and activating the activity of the NAD^+^-dependent enzyme, sirtuins. According to public safety assessments, the bioavailability and safety profile of NMN and NR are superior to those of other precursors, particularly of NR. The dose of NR that causes the lowest level of side effects is 1000 mg/kg/day, and the daily recommended dose of NR is 300 mg/kg. Hence, it does not produce side effects at the recommended dose [194]. Because the level of CD38 enzyme, which degrades NMN, increases with cell aging, NR may be more effective than NMN in elderly people. Therefore, in-depth studies on the safety, pharmacological effects, and side effects of NR, NMN, NA, and NAM are required to explore the NAD^+^ precursor most suitable as an exogenous supplement for NAD^+^.

Among all the NAD^+^ precursors, NMN and NR protect against metabolic disorders, cardiovascular diseases, and central nervous diseases in animal experiments. However, there are few clinical studies on the protective effects of NR and NMN on these diseases. In April 2021, the first randomized, double-blinded clinical trial evaluated the effect of NMN on the metabolic function of postmenopausal women with overweight or obesity [195]. The data showed that after 10 weeks of continuous oral administration of NMN (250 mg per day), the subjects' skeletal muscle insulin signal increased and insulin sensitivity improved. NMN upregulated platelet-derived growth factor (PDGF) receptor β and other genes related to skeletal muscle remodeling. This clinical study initially indicates that NMN can increase muscle insulin sensitivity in obese middle- aged or elderly women. In the future, more comprehensive clinical studies must be conducted to explore whether NMN and NR protect against metabolic disorders, cardiovascular diseases, and central nervous diseases in humans.

Although both NMN and NR protect against various diseases related to aging and even reverse the aging process in animals, the question that remains is can NMN and NR improve human age-related diseases or slow down the human aging progress? The questions that arise are: whether NMN or NR improves mitochondrial respiration and thus maintains the function of mitochondria in humans; whether NMN or NR delays or slows down the neurodegeneration progression in AD, PD, or other central nervous diseases in humans; and whether NMN or NR improves cardiovascular functions in heart failure or cardiac ischaemia in humans. Therefore, more studies are requiring before initiating their clinical applications.

Another debatable issue is the dosage of NAD^+^ precursors. The dose used in current research ranges from a few milligrams to 1 g/kg. From the pharmacological viewpoint, an appropriate dose has not been reasonably determined thus far. Therefore, determination of the appropriate dose and dosing frequency remains to be studied. Additionally, guidelines are required to monitor the beneficial effects and side effects of these precursors, if used as drugs in clinics or as food supplements, in humans. According to a study, the pharmacological effects of long-term supplementation of NAD^+^ precursors may change over time [79]. Thus, further research is required to investigate the NAD^+^ content that decreases in various tissues with age. We speculate that if it is a physiological decrease, whether it is the body's protective measure for reduced demand, or if the pathological decrease is caused by insufficient intake or excessive consumption, whether everyone requires exogenous NAD^+^ supplements during old age, and whether a high NAD^+^ content is favourable.

The NAD^+^ salvage pathway is also essential for immune cell functions, as discussed earlier, and NR can significantly improve the survival rate of immune-deficient mice. Therefore, future research should focus on balancing the positive role of inhibition of the salvage synthesis pathway in promoting tumour cell death and the negative role of it in maintaining the normal function of immune cells. In tumour cells, NAD^+^ is mainly synthesised through the salvage pathway. This pathway may provide a novel target for anticancer therapy. Therefore, investigating the effect of NAD^+^ levels on different types of tumour cells is essential.

As a nutritional supplement, NR is relatively safe and therefore has been developed as a dietary ingredient. Researchers have proposed that NR treatment can simulate the benefits of calorie restriction, which is the only known method for increasing the human lifespan [141]. However, NR is relatively unstable, and therefore, development of new NR products is essential to enhance its stability. In a recent human pharmacokinetic study, the level of NAD^+^ in human blood was found to increase by more than two times after a single administration of NR chloride (NRCl); however, NRCl is hydrolysed and degraded into NAM and sugars in the gastrointestinal fluid, which produces the antagonising effect of nicotinamide against NR [196]. Therefore, developing a method to optimise the preparation method, storage method, and route of administration of NRCl in order to increase its stability and prevent the formation and accumulation of NAM is essential. A membrane-coated form of NRCl, known as NIAGEN, is considered safe when used in food or as a dietary supplement, and its safety has also been evaluated in a series of preclinical studies [197]. Recently, NRH, a reduced form of nicotinamide riboside, was discovered that defines a new path for NAD^+^ biosynthesis, which is NRK independent. NRH exhibits high bioavailability and an unprecedented ability to increase the level of NAD^+^. It increases the intracellular NAD^+^ content by 5- to 10-fold compared with the basal level in different cell lines and mice, and thus, it is more potent than NR [198]. Moreover, NRH is not degraded to NAM in plasma, which is an advantage of using NRH for NAD^+^ synthesis [59].

In conclusion, NAD^+^ precursors may offer benefits to human health; however, more studies are required to determine the dose-response relationship, pharmaceutical formulation, pharmacological actions, adverse effects, and particularly the long-term safety of NMN and NR. Necessarily, owing to the availability of limited information, the beneficial effects of NAD^+^ precursors should not be exaggerated.
